# A Systematic Review of Root Canal Filling Materials for Deciduous Teeth: Is There an Alternative for Zinc Oxide-Eugenol?

**DOI:** 10.5402/2011/367318

**Published:** 2010-10-17

**Authors:** Fernanda Barja-Fidalgo, Michele Moutinho-Ribeiro, Maria Angelina Amorim Oliveira, Branca Heloísa de Oliveira

**Affiliations:** Department of Community and Preventive Dentistry, Faculty of Dentistry, Rio de Janeiro State University, Avenida 28 de Setembro, 157, sala 226, 20551-030 Rio de Janeiro, RJ, Brazil

## Abstract

The aim of this systematic review was to determine whether there is a root canal filling for deciduous teeth equally or more effective than zinc oxide-eugenol cement (ZOE). Six clinical trials selected for inclusion were independently reviewed by two researchers. Only two showed statistically significant different success rates between the test and the control groups. One found that an iodoform paste with calcium hydroxide (IP + Ca) performed better than ZOE, and the other found that ZOE performed similarly to IP + Ca. The other four studies compared ZOE with an iodoform paste (IP), a calcium hydroxide cement (Ca(OH)_2_), or IP + Ca. In these trials, the success rates in the ZOE groups were slightly lower than in the other groups. There seems to be no convincing evidence to support the superiority of any material over ZOE, and both ZOE and IP + Ca appear to be suitable as root canal fillings for deciduous teeth.

## 1. Introduction

Pulp therapy for deciduous teeth aims to preserve the child's health and to maintain deciduous teeth where pulp tissue is affected by caries, dental trauma, or other causes in a functional state until they are replaced by permanent teeth [1]. When the pulp has become irreversibly infected or necrotic, a root canal treatment is indicated [2, 3]. However, the complex morphology of the root canal system in deciduous teeth makes it difficult to achieve proper cleansing by mechanical instrumentation and irrigation of the canals [4]. So, in order to increase the chance of success of the endodontic treatment, substances with antimicrobial properties are frequently used as root canal filling materials in deciduous teeth.

Zinc oxide-eugenol cement (ZOE) has long been used as a root canal filling material for deciduous teeth [5], and in a survey conducted in 1997 it was cited as the preferred root canal filling material by 94% of the chairpersons of predoctoral pediatric dental programs in the Unites States [6]. Nevertheless, ZOE cannot be considered the ideal root canal filling material because it presents limited antimicrobial action [7] and it tends to resorb at a slower rate than the roots of the deciduous teeth [8, 9]. Concerns about these shortcomings of ZOE led to a search for alternative root canal filling materials for deciduous teeth (e.g., pastes containing iodoform, calcium hydroxide, or both).

Iodoform pastes have better resorbability and disinfectant properties [1, 6, 8] than ZOE, but they may produce a yellowish-brown discoloration of the tooth crowns which may compromise esthetics [1]. Different formulations of root canal filling materials containing iodoform are available: Kripaste (iodoform, camphor, menthol, and parachlorophenol), Maisto paste (iodoform, camphor, menthol, parachlorophenol, zinc oxide, lanolin, and thymol), Guedes-Pinto paste (iodoform, camphorated parachlorophenol, and Rifocort (Medley, Campinas/SP; prednisolone acetate and sodium rifampicin)), Endoflas (Sanlor Lab, Miami/FL; iodoform, zinc oxide, calcium hydroxide, barium sulfate, eugenol, and paramonochlorophenol), and Vitapex (Neo Dental International, Federal Way/WA; calcium hydroxide and iodoform) [10, 11].

Calcium hydroxide is considered to have some antimicrobial action and is easily resorbed when inadvertently forced beyond the dental apex [12, 13]. The antimicrobial action of calcium hydroxide is associated with its ionic dissociation into Ca_2_^+^ and OH^−^ ions. The vehicle used in the formulation of the root canal filling paste plays a fundamental role in this process because it influences the speed of ionic dissociation. Generally, three types of vehicles are used: aqueous, viscous, and oily [14]. Aqueous vehicles favor a high degree of solubility, causing the paste to be completely removed from within the root canals before the time of physiological tooth replacement, requiring new endodontic interventions to be undertaken [9]. Viscous vehicles promote a lower solubility of the paste in comparison to aqueous vehicles, and oily vehicles promote the lowest solubility and diffusion of calcium hydroxide pastes. Pastes containing an oily vehicle, particularly those with an antibacterial substance (i.e., iodoform) have shown more favorable results than more soluble pastes, when used as a root canal filling material in primary teeth [15].

Numerous techniques and root canal filling materials for the endodontic treatment of deciduous teeth are described in the dental literature [9, 16–19] but there is little agreement between pediatric dental educators as to the best available treatment procedures [6, 20]. 

The aim of this research was to determine, through a critical evaluation of the dental literature, whether there is a root canal filling material for endodontically treated deciduous teeth equally or more effective than ZOE.

## 2. Methods

### 2.1. Study Design

Systematic review of the dental literature.

### 2.2. Inclusion Criteria

To be included in the present review, an article had to meet the following criteria.

- Study design: randomized, quasi-randomized (i.e., the method used for allocating people to the trial was not random but was intended to produce similar groups, such as allocation by the person's date of birth or just allocating every other person) [21], or controlled clinical trial;
- participants: children of any age;
- type of intervention: pulpectomy in deciduous teeth, using ZOE as a root canal filling material in one group (control group) and another dental material (e.g., iodoform paste or calcium hydroxide paste) in the other group (test group);
- outcome measure: clinical and/or radiographic success rate at the end of the followup period;
- language: English, Spanish, or Portuguese.

### 2.3. Search Strategy

Searches of the MEDLINE database were performed during the months of June and July 2009 using various combinations of the following terms: “pulp therapy,” “pulpectomy,” “endodontic treatment,” “endodontic therapy,” “root canal treatment,” “necrosis,” “iodoform,” “maisto,” “guedes-pinto,” “vitapex,” “kripaste,” “3mix,” “metronidazole,” “ornidazole,” “formocresol,” “CTZ” (root canal filling material that contains chloramphenicol, tetracycline and zinc oxide-eugenol), “tetracycline,” “chloramphenicol,” “ciprofloxacin,” “minocycline,” “antibiotics,” “parachlorophenol,” and “deciduous teeth”. Two reviewers read the titles and abstracts of the 947 articles that were found in order to determine whether they were eligible for inclusion. Duplicate papers and those that did not fulfill the inclusion criteria were eliminated, and, eventually, fifteen papers were identified as potentially relevant. Full reports of these articles were retrieved, and final selection was made independently by the same two reviewers based on screening of the contents using a previously prepared data-extraction form. Discrepancies were resolved by involving a third reviewer. A hand search of the reference lists of these 15 papers was performed, and no other paper was identified through this strategy. The excluded papers [1, 16–19, 22–25] and the reasons for their exclusion are depicted in Table 1. 

Subsequently, the Cochrane database was searched, and one relevant systematic review was identified [26]. Its reference list was examined but no additional eligible studies were found. 

### 2.4. Quality Assessment

Critical appraisal of the included studies was done independently by two reviewers based on a set of items considered key to the study outcomes [27, 28] (Table 2). Any disagreements between the reviewers were resolved by reaching a consensus, and, when necessary, differences were resolved by a third reviewer.

## 3. Results

Of the six studies included in the present review, one was from Iran [8], one was from Turkey [9], one was from Thailand [29], and three were from India [13, 30, 31]. In these clinical trials, 352 deciduous teeth (339 posterior teeth and 13 anterior teeth) of 312 children aged 3 to 13 years were endodontically treated and followed for periods ranging from 6 to 18 months (Table 3).

Two studies compared ZOE with a calcium hydroxide paste [13, 30], two studies compared ZOE with a premixed calcium hydroxide and iodoform paste (Vitapex) [8, 29], one study compared ZOE with a zinc oxide/iodoform paste (Maisto paste) [31], and one study compared ZOE with three different root canal fillings: two calcium hydroxide-based materials: Sealapex—a calcium hydroxide cement—and Calcicur—a calcium hydroxide paste—and one calcium hydroxide and iodoform paste (Vitapex) [9]. The main characteristics of the included studies are depicted in Table 3.

Five trials [8, 9, 29–31] stated that random allocation was used to assign participants to the comparison groups, and one of them [8] described the use of a proper random allocation procedure. In four studies [9, 29–31], the unit of randomization was the tooth, not the child, and this meant that there was more than one observation per participant. Two trials [8, 31] included anterior and posterior teeth whereas four trials [9, 13, 29, 30] included only deciduous molars.

None of the experiments were described as double blind but in two of them [9, 29] it was reported that the clinical and radiographic assessments of treatment outcomes were performed by blinded examiners. In only one study [8] were participants lost to followup (around 10%) but information about the number of participants that did not complete the study in each group was not provided. 

The trials reported similar inclusion and exclusion criteria but used different treatment techniques, especially in relation to the number of appointments and to the type of root canal irrigating solution and restorative material (Table 4). None of the reviewed articles reported how sample size was calculated. Study findings were assessed in terms of their statistical significance, and two articles [8, 29] provided actual *P* values. In five trials [9, 13, 29–31], multiple observations from each participant were treated as independent. The studies used similar criteria for the assessment of clinical success. Differences were noticed in terms of how radiographic success was defined. Mani et al. [13], Mortazavi and Mesbahi [8], and Trairatvorakul and Chunlasikaiwan [29] considered treatment to have been successful when a decrease in the size of a previously existent radiolucent area was observed. However, the latter [29] classified teeth without improvement in the radiolucency by 6 months as requiring “further observation”. Nadkarni and Damle [30] considered a case to be successful if a radiolucency observed at the baseline did not increase, and Özalp et al. [9] and Reddy and Fernandes [31] considered as successful only cases without a radiolucency in the furcal or periradicular areas. In all trials, success rates were equal to or above 78% (Table 3).

## 4. Discussion

The lack of treatment of a deciduous tooth with irreversible pulpitis or pulpal necrosis can cause damage to the succedaneous tooth (e.g., enamel hypomineralization or hypoplasia) [32] and produce negative impacts on the child's oral health-related quality of life (e.g., pain, missed school days and difficulty in chewing) [33]. Therefore, teeth presenting these conditions should be extracted or subjected to root canal treatment [4].

Various techniques for the endodontic treatment of deciduous teeth have been described [18, 19, 34, 35]. Traditionally, ZOE has been the material of choice for filling the root canals of deciduous teeth [36], and until 2008 it was the only material explicitly recommended in the clinical guidelines developed by the American Academy of Pediatric Dentistry (AAPD) [2]. In 2009, based on studies recently published, the AAPD guidelines began to cite iodoform-based pastes as suitable alternatives to ZOE [3]. In addition, the results of a survey of diplomates of the American Board of Pediatric Dentistry and US predoctoral pediatric dental program directors conducted in 2005 showed that significantly fewer diplomates and directors advocated ZOE for root canal filling when compared to directors surveyed in 1997. This may be due to concerns about the possible detrimental effects of residual ZOE filler particles on the succedaneous teeth [20].

Only two of the six studies included in the present review reported significant statistical differences between the frequency of successful cases in the test and control groups at the end of the followup period [8, 9]. However, in all of the studies, sample sizes were small and this may have resulted in a very low power to detect clinically meaningful treatment effects. The success rates of the two trials [8, 31] that included anterior teeth were similar to the success rates of the trials that included only posterior teeth. In only one study [9] the type of tooth was considered in the analysis, and no statistically significant differences between the success rates of endodontic treatment performed in upper and lower molars were found. 

One trial [8] found that Vitapex was more effective than ZOE because it produced a greater decrease in abnormal tooth mobility and in pre-existent bone radiolucency. Furthermore, at the end of the followup period, no evidence of extruded filling material was observed in the Vitapex group whereas particles of extruded material had not changed in size in a few patients of the ZOE group. The other study [9] concluded that both ZOE and Vitapex were 100% successful, but in the Vitapex group, six teeth needed retreatment because of complete resorption of the root canal filling material, and in the ZOE group, of the six teeth overfilled, only two showed complete resorption of the extruded material. In the same study, the success rates of ZOE and a calcium hydroxide paste were compared, and ZOE performed significantly (*P* < .05) better than the root canal filling material containing calcium hydroxide. 

One study found significant statistical differences between the frequency of radiographic success in the test and control groups, at the 6-month followup, but by 12 months this difference had disappeared [29].

The six trials included adopted similar criteria for inclusion and exclusion of participants and used the same definition of clinical success. However, the definitions of radiographic success were so diverse that cases considered successful in one study [30] would be classified as failures by the standards employed by two other studies [9, 31]. All six studies were also heterogeneous in terms of treatment techniques (i.e., number of appointments, irrigating solutions, and type of tooth restoration). In one [13] of the studies the materials used to make the final restorations did not provide an adequate barrier against bacterial penetration and it has been shown that the absence of proper coronal sealing is associated with the failure of endodontic treatment [35, 37]. Pooling the results of the included studies was thus considered inappropriate.

Potential threats found to the internal validity of the studies are also worth mentioning. In clinical trials, the comparability between the test and control groups, in relation to factors other than the intervention that might influence the outcome, is crucial and is usually obtained through an adequate process of randomization in treatment allocation. Only one study properly randomized patients to treatment groups and also included just one tooth per child [8]. However, the characteristics of the treatment groups at baseline were not described in the article, thus limiting one's ability to assess the success of the randomization process. Other features of the included studies, such as lack of masking and calibration of the examiners, may also have biased their results. Equally important is the fact that three [13, 30, 31] of the selected studies had less than one year of followup.

The Cochrane review [26], published in 2003, provided a reasonably comprehensive picture of the state of the art, but it is important, seven years out, to again raise a red flag about the lack of studies with acceptable methodological quality.

Altogether, the results of this critical appraisal of the studies indexed in the Medline database comparing ZOE with other root canal filling materials for deciduous teeth have shown that randomized clinical trials designed as recommended by the Consolidated Standards for Reporting Trials (CONSORT) [27] are lacking in this field. Future studies should also seek to address long-term effects of treatment (i.e., damage to dental enamel or deflection of succedaneous tooth) and choose outcome measures clinically meaningful to the patients and their caregivers (i.e., premature extraction of deciduous teeth, a need for dental restoration in the successor tooth or a need for orthodontic treatment).

## 5. Conclusion

There seems to be a moderate level of evidence to support the use of both ZOE and iodoform paste—with calcium hydroxide—as root canal filling materials for deciduous teeth. High-quality randomized controlled clinical trials with at least 12 months of followup are necessary before a reliable conclusion can be drawn as to the best root canal filling material for endodontically treated deciduous teeth.

## Figures and Tables

**Table 1:** Reasons for the exclusion of eight articles read in full.

Reference	Reason for exclusion
Garcia-Godoy, 1987 [1]	No comparison group
Sogbe de Agell, 1989 [16]	Did not test ZOE
Flaitz et al., 1989 [23]	No comparison group
Holan and Fuks, 1993 [18]	Observational study
Nurko and Garcia-Godoy, 1999 [17]	No comparison group
Takushige et al., 2004 [19]	No comparison group
Damle and Nadkarni, 2005 [22]	Reported the results of a study previously selected
Önçag et al., 2006 [24]	Did not test a root canal filling material
Coser and Giro 2008 [25]	Tested ZOE in pulpotomy, not in pulpectomy

**Table 2:** Criteria used for critical appraisal of the included studies.

Criteria	Description
Participants	Criteria used for inclusion of participants in the study and settings and locations where the data was collected
Sample size	How sample size was estimated
Equivalence of study groups at baseline	Baseline demographic and clinical characteristics of trial groups
Outcomes	Clinical and radiographic criteria used to evaluate treatment success; methods used to enhance the quality of measurements
Intervention	Detailed description of the interventions administered for each group (i.e., ZOE group and comparison group using another root canal filling material)
Followup	Duration of followup; description of protocol deviations
Statistical analysis	Methods used to compare groups for primary outcome
Randomization	Assignment of participants to the groups in the trial by a method the uses the play of chance
Blinding	Process of preventing those involved in the trial from knowing to which comparison group a particular participant belongs
Withdrawals	Description of characteristics of participants lost to followup

**Table 3:** Main characteristics and results of the clinical trials included in this review.

Reference	Allocation sequence generation	Root canal filling	Sample	Followup period in months (recall interval)	Success rate (comparison group) *n* (%)	Success rate (ZOE group) *n* (%)	*P* value	Withdrawals *n* (%)
Reddy and Fernandes 1996 [31]	Randomization by tooth	Maisto paste	30 teeth (1 upper molar, 21 lower molars, and 8 upper anterior teeth) 26 children 3–8 years	9 (3–6 and 9)	15 (100.0)	12 (80.0)	n.r.*	0

Mani et al. 2000 [13]	Randomization not mentioned	Pulpdent	60 teeth (21 1st lower molars, and 39 2nd lower molars) 50 children 4–9 years	6 (2)	26 (86.7)	25 (83.3)	n.s.^†^	0

Nadkarni and Damle 2000 [30]	Randomization by tooth	Calcium hydroxide paste	70 teeth (19 1st lower molars 51 2nd lower molars) 60 children 4–8 years	9 (3)	33 (94.3)	31 (88.6)	n.s.^†^	0

Mortazavi and Mesbahi 2004 [8]	Randomization by child	Vitapex	58 teeth (23 upper molars, 30 lower molars, and 5 upper anterior teeth) 58 children 3–13 years	10–16 (3)	n.r.* (100.0)	n.r.* (78.5)	*P* = .015	6 (10)

Özalp et al. 2005 [9]	Randomization by tooth	Calcicur Sealapex Vitapex	80 teeth (23 1st upper molars, 32 2nd upper molars, 14 1st lower molars, and 11 2nd lower molars) 76 children 4–9 years	18 (2)	16 (80.0) 18 (90.0) 20 (100.0)	20 (100.0)	*P* < .05 n.s.^†^ n.s.^†^	0

Trairatvorakul and Chunlasikaiwan 2008 [29]	Randomization by tooth	Vitapex	54 teeth (12 1st left lower molars, 10 1st right lower molars, 13 2nd left lower molars, and 19 2nd right lower molars) 42 children 3–7 years	12 (6)	24 (89.0)	23 (85.0)	*P* = 1 n.s.^†^	0

n.r.*: not reported; n.s.^†^: not significant

**Table 4:** Characteristics of the techniques for deciduous tooth pulp therapy used in the studies included in this review.

	Reference					
Clinical procedures	Reddy and Fernandes (1996) [31]	Mani et al. (2000) [13]	Nadkarni and Damle (2000) [30]	Mortazavi and Mesbahi (2004) [8]	Özalp et al. (2005) [9]	Trairatvorakul and Chunlasikaiwan (2008) [29]
Number of appointments	Two or more. 1st: pulpotomy plus formocresol dressing. 2nd: canal cleansing and obturation. In case of abscess, dressings were repeated till tooth was asymptomatic.	One or more. Teeth with abscess were opened for drainage and a course of antibiotics was prescribed prior to root canal treatment. Uncooperative children also received a two-sitting procedure.	One.	Two. 1st: pulpotomy plus formocresol dressing. In case of abscess, a course of antibiotics was prescribed. 2nd: canal cleansing and enlargement plus filling.	One or two. Only uncooperative children received a two-sitting procedure.	One.

Type of root canal irrigating solution	Diluted hydrogen peroxide (3%) and saline solution.	5% sodium hypochlorite and 0.5% metronidazole solution.	2.5% sodium hypoclorite and saline solution.	Saline solution.	5% sodium hypoclorite and 0.5% metronidazole solution.	2.5% Sodium hypoclorite.

Root canal instrumentation	Up to file number 20.	Up to Hedstrom file 30–35.	Up to Hedstrom file 40.	Two or three sizes greater than the first file (beginning with K-file 15).	Up to Hedstrom file 30–35.	Up to K-file 35–40.

Type of dental restoration	Stainless steel crown.	Fast-setting ZOE cement.	Stainless steel crown.	Silver amalgam or composite resin.	Silver amalgam.	Stainless steel crown.
